# Mental retardation in younger children

**DOI:** 10.1186/1755-8166-7-S1-P89

**Published:** 2014-01-21

**Authors:** Grishma Shukla, Swati B Thakur, GT Swamy, MV Rao

**Affiliations:** 1 Gujarat Genetic Diagnostic Center (GenDiCe), Department of Zoology, University School of Sciences, Gujarat University, Ahmedabad, Gujarat, India; 2 B M Institute of mental health, Ahmedabad-09, Gujarat, India

## Background

It is a generalized disorder appearing before adulthood, characterized by significantly impaired cognitive functioning and deficits in two or more adaptive behaviours. The present study was aimed to investigate the delayed development and psychological aspects in symdromic and non syndromic mental retarded individuals.

## Material and methods

This study was conducted at B.M. Institute, Ahmedabad. The aetiology was ascertained after proforma studying and number of patients observed. The spectrum of causative conditions and contribution of genetic disorders was established. A total of 40 patients were observed in which 30 were male & 10 were female between the ages of 3 -11 years. The various aetiological categories were: chromosomal disorders in 17, non chromosomal syndromes in 23. In which chromosomal disorders include Down syndrome, non-chromosomal disorders include delayed development and cerebral palsy.

## Results

Among non syndromic mental retardation (NSMR) children; the walking is delayed by 1 yr in 22% of children, 2 yrs in 57 % of children, 3 yrs in 4% and 13 % children were not able to walk. In syndromic mental retardation (SMR) walking is delayed by 1 yr in 41 % of children, 2 yrs in 23 % of children, 3 yrs in 24 %, 6 % were not able to walk. Other developmental milestone such as sitting and speech in both syndromic and non syndromic mental retardation also showed the same pattern of delayed development as seen in the walking. In IQ, level the highest is SMR which is 44% severe & 45% moderate while in NSMR 31% severe & 38% moderate level were observed.

## Conclusions

The present investigation concluded that there was a delayed milestone in mental retarded children which was supported by IQ levels in this study.

